# Application of Micromechanical Modelling for the Evaluation of Elastic Moduli of Hybrid Woven Jute–Ramie Reinforced Unsaturated Polyester Composites

**DOI:** 10.3390/polym13152572

**Published:** 2021-08-01

**Authors:** Agung Efriyo Hadi, Mohammad Hazim Mohamad Hamdan, Januar Parlaungan Siregar, Ramli Junid, Cionita Tezara, Agustinus Purna Irawan, Deni Fajar Fitriyana, Teuku Rihayat

**Affiliations:** 1 Mechanical Engineering Department, Faculty of Engineering, Universitas Malahayati, Jl. Pramuka No. 27, Kemiling, Bandar Lampung 35153, Indonesia; 2 Faculty of Engineering and Computing, First City University College, No. 1, Persiaran Bukit Utama, Bandar Utama, Petaling Jaya 47600, Malaysia; 3 College of Engineering, Universiti Malaysia Pahang, Gambang 26300, Malaysia; ramli@ump.edu.my; 4 Department of Mechanical Engineering, Faculty of Engineering and Quantity Surveying, INTI International University, Nilai 71800, Malaysia; 5 Faculty of Engineering, Universitas Tarumanagara, Jakarta 11480, Indonesia; agustinus@untar.ac.id; 6 Department of Mechanical Engineering, Universitas Negeri Semarang, Kampus Sekaran, Gunungpati, Semarang 50229, Indonesia; deniifa89@mail.unnes.ac.id; 7 Departement of Chemical Engineering, Politeknik Negeri Lhokseumawe, Aceh 24301, Indonesia; teukurihayat@yahoo.com

**Keywords:** natural fibre, jute fibre, ramie fibre, micromechanical, polymer

## Abstract

Woven laminated composite has gained researchers’ and industry’s interest over time due to its impressive mechanical performance compared to unidirectional composites. Nevertheless, the mechanical properties of the woven laminated composite are hard to predict. There are many micromechanical models based on unidirectional composite but limited to the woven laminated composite. The current research work was conducted to evaluate elastic moduli of hybrid jute–ramie woven reinforced unsaturated polyester composites using micromechanical effectiveness unidirectional models, such as ROM, IROM, Halpin–Tsai, and Hirsch, which are based on stiffness. The hybrid jute–ramie laminated composite was fabricated with different layering sizes, and the stacking sequence was completed via hand lay-up with the compression machine. Tensile modulus values for hybrid composites are between those for single jute and single ramie. Obtained *p*-values less than 0.05 prove the relationship between layering size and tensile modulus. This study showed that several micromechanical models, such as Halpin–Tsai’s predicted value of homogenized mechanical properties, were in good agreement with the experimental result. In the case of the hybrid composite, the micromechanical model deviates from the experimental result. Several modifications are required to improve the current existing model. A correlation function was calculated based on the differences between the elastic modulus values determined experimentally and those derived from each micromechanical model calculation.

## 1. Introduction

The reinforcement fibres were incorporated into the matrix polymer to increase the material’s tensile strength and stiffness, resulting in high-performance materials. The researchers incorporated a variety of reinforcement types, including discontinuous and continuous fibres. While discontinuous fibres are isotropic, they generate less strength in one direction than continuous fibres. As a result, the researcher chose continuous fibre as reinforcement material to achieve greater axial strength for the composite. The laminate geometry is a frequently used geometry for continuous fibre composites. Laminated composites are formed by combining two or more sheets of reinforcing material in a matrix polymer [1]. Laminated composites are interesting because they offer significant mechanical performance and are lightweight, making them well-suited for automotive, aeronautic, sporting, and marine applications [1,2].

Researchers have employed various laminate fabrics, such as unidirectional, woven, nonwoven, knitted, and braided laminate fabrics [3,4,5] as reinforcement fabric to solve engineering problems. Among all of the laminated fabric types, many researchers have reported using woven structures as reinforcement materials. For example, a woven fabric is known for its many advantages over unidirectional fabric. Woven composite is easy to handle and highly resistant to impact [6]. Due to the yarn fibres in the warp and weft directions, a woven fabric is stable. It can distribute the load in any longitudinal or transverse direction while retaining its original shape or dimensions [7]. Moreover, the woven fabric is interesting because it is easy to handle during the fabrication process. Most woven fabric is limited to the perpendicular angles, 0° and 90°. Different types of woven fabric are available in the current market, such as plain, twill, satin and basket [8]. Among all, woven fabric with plain architecture is the simplest and most commonly used by researchers in laminated composites. Woven laminated composite provides better out-of-plane stiffness and excellent mechanical properties such as tensile, flexural and impact [9].

Natural fibre has been used as reinforcement material for petroleum-based and renewable matrix polymer to form a partial green composite or full green composite materials. Green composited is a term coined from bio-based plastic and natural fibre [10]. Natural fibre-based composites provide an environmentally sustainable alternative to synthetic fibre. Previously published research has examined the sustainability of natural fibre and its composites as reinforcement materials through a life cycle assessment (LCA) analysis, which examines the impact of each material’s life cycle stage [11,12]. By substituting natural fibres for synthetic fibres, particularly in automotive and structural applications, a higher level of light-weighting can be achieved with several environmental benefits, including recyclability, renewability, and lower material costs. Additionally, unlike synthetic fibre, natural fibre composites are biodegradable, degrading completely when discarded into the environment [12,13]. To date, many types of natural fibre have been applied by researchers, such as jute [5], ramie [14,15], kenaf [8] and roselle [16]. However, there are several issues regarding natural fibre, such as inconsistent fibre dimensions and chemical composition.

The determination of mechanical properties for natural woven fibres is vital for designing new laminated structures. Mechanical behaviour and elastic properties for the natural woven laminated composite are hard to predict, leading to extensive labour, time-consuming processes and increased cost. There are numerous parameters involved for natural woven laminated composite, which are woven parameters and laminated parameters. Woven parameters consist of yarn crimp, yarn twist, linear density, and fabric architecture [17]. Meanwhile, laminated parameters consist of layering size, layering sequence, fabric orientation, and volume/weight fraction [18]. A combination of these aspects makes modelling for woven laminated composite extremely challenging compared to the continuous fibre. Researchers have proposed several micromechanical models to determine the in-plane properties of woven laminates analytically.

Many micromechanical models are based on unidirectional composites, such as the rule of mixture (ROM), inverse rule of mixture (IROM), Halpin–Tsai, and Hirsch. In this study, the tensile modulus of single jute, single ramie and hybrid jute–ramie reinforced unsaturated polyester composite was determined using experimental analysis and the micromechanical models such as ROM, IROM, Halpin–Tsai and Hirsch. The values obtained from the experimental analysis and micromechanical models were compared via graphs to verify which theory values are very close to the experimental values.

## 2. Materials and Methods

### 2.1. Materials

The main reinforcement materials used in the current study were jute and ramie. Jute and ramie fibre are bast fibres. Jute is a very affordable natural fibre, with high tensile strength, low thermal conductivity, and moderate moisture regain [19]. Ramie fibre is among the strongest natural fibres, with high tenacity and good breaking extension in dry and wet conditions [20]. The woven jute and ramie is a form of 1/1 plain-woven fabric. The jute and ramie fibre is processed and supplied by Indonesia (Craft Material Center, Jogjakarta, Indonesia) The specification for the woven jute and ramie is presented in Table 1.

The unsaturated polyester (UPE) Reversol P-9539 NW by manufacturer Revertex (Johor, Malaysia) was selected as matrix polyester due to its low cost, acceptable mechanical properties, high corrosion resistance and low weight; it was supplied by Impian Enterprise (Kuala Lumpur, Malaysia). The UPE resin has a specific gravity of 1.1 to 1.2. The tensile strength and tensile modulus for pure UPE resin are 33–104 MPa and 2-4 GPa, respectively. Methyl ethyl ketone (MEKPO) is used as a hardening agent.

### 2.2. Methodology

Fabrication of the unsaturated polyester composite with woven jute–ramie reinforcement was accomplished by hand lay-up and compression. Squares of plain-woven jute and ramie fabrics with dimensions of 300 mm × 300 mm were cut. The ratio of unsaturated polyester to MEKPO was then determined using an analytical balance at a ratio of 1:50. A release agent was sprayed on the mould’s surface to facilitate the removal of the composite plate after curing. The woven fabric was placed on top of the mould. The UPE mixture was then poured onto the woven fabric. The roller was used to remove excess air from the fabric’s surface and ensure uniform resin distribution. The design of the jute–ramie reinforced, laminated composite with varying layering sizes and stacking sequences is summarised in Table 2. The table also includes a breakdown of the total fibre and matrix weights used. According to Table 2, the jute fabric and ramie were designated as ‘J’ and ‘R’, respectively. For instance, the first letter in JJRR indicates that the fabric is at the top, while the final letter indicates that it is at the bottom.

The mould was tightly closed and subjected to a load of between 100 and 200 N. At a room temperature of 22–24 °C, curing takes approximately 8 h. Following that, the fabricated composite was cut into a tensile testing specimen in accordance with ASTM 3039. The composite sample has dimensions of 25 mm (length) × 25 mm (width) × 3 mm (thickness). Tensile testing was performed using a universal testing machine (UTM) (INSTRON 3369, Mechanic of Materials Laboratory, Universiti Malaysia Pahang, Malaysia) equipped with a 10 kN load cell. It was performed on the UTM machine at a crosshead speed of 1 mm/min. The fabric was tested in the direction of the warp. Each group received seven specimens. Statistical analysis (ANOVA) was performed on the data gathered during the testing.

### 2.3. Micromechanical Model

There are four (4) micromechanical models that were applied to the current research work, which consist of the rule of mixture (ROM), inverse rule of mixture (IROM), Halpin–Tsai and Hirsch.

#### 2.3.1. Rule of Mixture (ROM) and Inverse Rule of Mixture (IROM)

The rule of mixture (ROM) is a fundamental way to calculate and predict the mechanical performance of composite materials. The rule of the mixture can be modified based on the fibre alignment. The ROM equation is presented as follows:

(1) $$E_{c}=E_{f}V_{f}+E_{m}V_{m}$$

e *E* is Young’s modulus, while *V* is a volume fraction. Variables *c*, *f*, and *m* represent composite, fibre and matrix, respectively.

Nonetheless, there are several drawbacks to the ROM. Based on the literature, the ROM of the mixture is unable to predict the tensile performance accurately due to many factors such as stress and strain raisers due to embedded reinforcements, interface failure, statistical dispersion effect, void presence, and misalignment [21]. As a result of inaccuracies, the value of ultimate tensile strength obtained from the ROM is often more significant than the actual values. As assumed by ROM, the fibre in the composite is unidirectionally aligned and uniformly distributed. However, in reality, the fibre exhibits some non-homogeneity in its spread and is misaligned in its orientation. Additionally, the ROM does not aid in predicting the continuous fibre’s transverse direction. As a result, Equation (2) incorporates the inverse rule of mixtures (IROM). On the other hand, Equations (3) and (4) show the ROM and IROM for hybrid composites.

(2) $$E_{c}=E_{f}V_{f}+E_{m}V_{m}$$

(3) $$E_{1}=E_{f1}V_{f1}+E_{f2}V_{f2}+E_{m}V_{m}$$

(4) $$E_{2}=\frac{E_{m}E_{f1}E_{f2}}{V_{m}(E_{f1}E_{f2})+V_{f1}(E_{m}E_{f2})+V_{f2}E_{m}E_{f1})}$$

#### 2.3.2. Halpin-Tsai (H-T)

The Halpin–Tsai model is a mathematical model that uses the geometry and orientation of the filler, as well as the elastic properties of the filler and matrix, to predict the elasticity of composite materials. While also considered empirical, the model is based on the self-consistent field process. The Halpin–Tsai (H-T) is shown in Equations (5)–(9).

(5) $$M=\frac{M_{m}(1+\xi \eta V_{f})}{1-\eta V_{f}}$$

(6) $$\eta =\frac{E_{f}-E_{m}}{E_{f}+\xi E_{m}}$$

(7) $$M=\frac{M_{m}(1+\xi (\eta_{f1}V_{f1}+\eta_{f2}V_{f2}))}{1-(\eta_{f1}V_{{}_{f1}}+\eta_{f2}V_{f2})}$$

(8) $$\eta_{f1}=\frac{(\frac{E_{f1}}{E_{m}})-1}{(\frac{E_{f1}}{E_{m}})+\xi }$$

(9) $$\eta_{f2}=\frac{(\frac{E_{f2}}{E_{m}})-1}{(\frac{E_{f2}}{E_{m}})+\xi }$$

Equations (5) and (7) are composite moduli, including *E*_11_, *E*_22_, *G*_12_, *G*_23_, etc. Here, *V* is a volume fraction. The notations of *f* and *m* represent fibre and matrix, respectively, while *η* is a function.

The numbers 1 and 2 for fibre parts cover different types of fibre reinforcement. Moreover, the *ξ* denotes an empirical parameter or curve fitting parameter. It was used to ensure that the calculated value matches the experimental data. Nielsen (1974) highlighted that the value depends on the fibre packing arrangement, array type, hexagonal and square [22]. For the small value of *ξ* (*ξ* → 0), the Halpin–Tsai model will be reduced to a series model as in Equation (1). Otherwise, for the immense value of *ξ* (*ξ* → ∞), the Halpin–Tsai model will reduce the parallel model as in Equation (2). In this study, the empirical parameter with a value of 1 and 2 was selected. According to Adam and Doner (1967), *ξ* = 2 gives an excellent fit to the finite difference elasticity for the transverse modulus of a square array of circular fibre [23]. The value for this study’s empirical parameters is 1 and 2 for a single and a hybrid composite, respectively.

#### 2.3.3. Hirsch Model (HI)

Hirsch model is a model based on a parallel and series of the rule of mixture. It is more prevalent for the matrix material’s random-oriented fibre than other micromechanical models found based on the literature study. In the Hirsch model, the critical parameter is the stress transfer between the fibre and matrix, as shown in Equation (10):

(10) $$M_{c}=\kappa (M_{m}V_{m}{+M}_{f}V_{f})+(1-\kappa )\frac{M_{m}\times M_{f}}{M_{m}V_{f}{+M}_{f}V_{m}}$$

where M in Equation (10) is composite moduli, including *E*_11_, *E*_22_, *G*_12_, *G*_23_, etc. The notations *c*, *f* and *m* represent composite, fibre and matrix. The *κ* in Equation (10) is the stress transfer parameter, which varies between 0 and 1. The values of *κ* applied in previous research works consist of 0.01 [24], 0.1 [25] and 0.4 [26].

## 3. Result

### 3.1. Tensile Modulus for Single Fibre Composite

The tensile modulus values for single jute, single ramie, and hybrid jute–ramie reinforced fabrics are shown in Table 3 and Table 4. ANOVA was used to determine the relationship between layering size and tensile modulus. Table 5 summarises the ANOVA test results. The *p*-value obtained from the ANOVA was 0.00000284. When the *p*-value is less than 0.05, there is a significant relationship between layering size and tensile modulus.

### 3.2. Micromechanical Model for Single Fibre Composite

**Figure 1:**
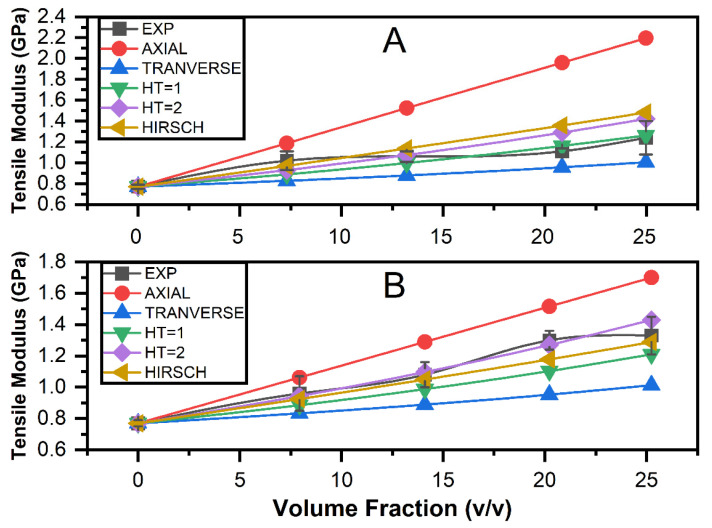
Modulus of versus volume fraction: (**A**) jute fibre composite (**B**) ramie fibre composite.

The experimentally determined modulus plot versus volume fraction for single jute and ramie fibre composite is shown in Figure 1. ROM, IROM, Halpin–Tsai, and Hirsch were used to calculate the theoretical strength of the composites. The elasticity modulus increases the fraction of the fibre length. Identical patterns can be found for both studies and theoretical values. However, the measured micromechanical model’s elasticity modulus value differed from the experimental one. As illustrated in Figure 1, the approximate values are comparable to the experimental value at the shorter fraction of the fibre length. However, at a fraction of the volume, there was a lot more variation. At higher volume fractions, it could be due to weak fibre–matrix interaction.

For both jute and ramie single fibre composites, modulus data increases as the volume fraction increases. The ROM measured in the axial direction yields higher values for all volume fraction ranges, as shown in Figure 1. The result demonstrates that the ROM in the axial direction produces upper bound results. The ROM in the transverse direction, on the other hand, yields the lower bound result. The ROM result in Figure 1 differs significantly from the experimental one. It was discovered that the hybrid mixture law predicts a higher bound in the hybrid system’s tensile properties. This matter is due to the hybrid system’s disregard for the influence of fibre orientation and fibre interaction with the matrix.

The elasticity modulus for empirical parameter *ξ* = 1 in the Halpin–Tsai micromechanical model shows an almost perfect agreement with data obtained from a single jute fibre composite in volume fractions ranging from 20% to 25%. The Halpin–Tsai micromechanical model with empirical parameter *ξ* = 1 does not fit the experimentally determined value for ramie fibre single composite. The Halpin–Tsai model with empirical parameter *ξ* = 2 only fits ramie fibre composite data with volume fractions ranging from 0% to 20%. For the most significant volume fraction of fibre, the accuracy of the Halpin–Tsai mathematical model improved significantly. As a result, it is possible to conclude that the Halpin–Tsai mathematical model can be used to provide an accurate result for woven natural fibre composite materials.

For a single jute composite, the Hirsch model only fits volume fractions ranging from 0% to 15%. There is a noticeable divergence in modulus of elasticity as the reinforcement volume fraction increases. The Hirsch model almost perfectly fits the experimental data for ramie fibre composite.

According to Table 6 and Table 7, a value of 0 indicates that the micromechanical model is close to the experimentally obtained value. A positive value indicates that the obtained value is greater than the experimentally obtained value. In contrast, a negative value indicates that the statistical model’s value is less than the experimental value.

### 3.3. Hybrid Fibre Composite

ROM, IROM, Halpin–Tsai, and Hirsch micromechanical models were used to calculate unsaturated jute–ramie hybrids’ overall stiffness or elasticity modulus. Depending on the layering size and sequence, the jute and ramie volume content in a jute–ramie hybrid composite ranges from 5% to 14%.

**Figure 2:**
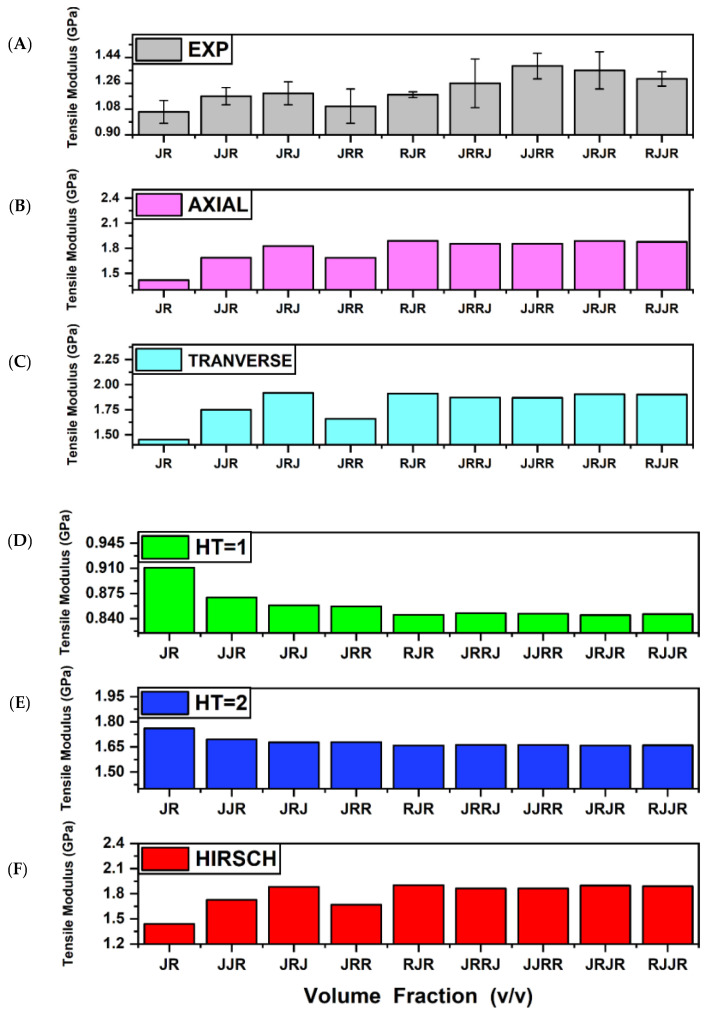
Modulus of composite hybrid: (**A**) Experiment (**B**) ROM (**C**) IROM, (**D**) HT = 1 (**E**) HT = 2. (**F**) HI.

The elasticity modulus of a jute–ramie hybrid composite is shown in Figure 2 using the ROM, IROM, Halpin–Tsai, and Hirsch micromechanical models. According to Figure 2, none of the micromechanical models fit the experimentally determined value. The IROM and Hirsch models yield the highest theoretical value derived from empirical 1 in the H-T. Nonetheless, the calculated values from Halpin–Tsai and Hirsch are nearly identical, particularly for the high volume fraction of fibre.

**Figure 3:**
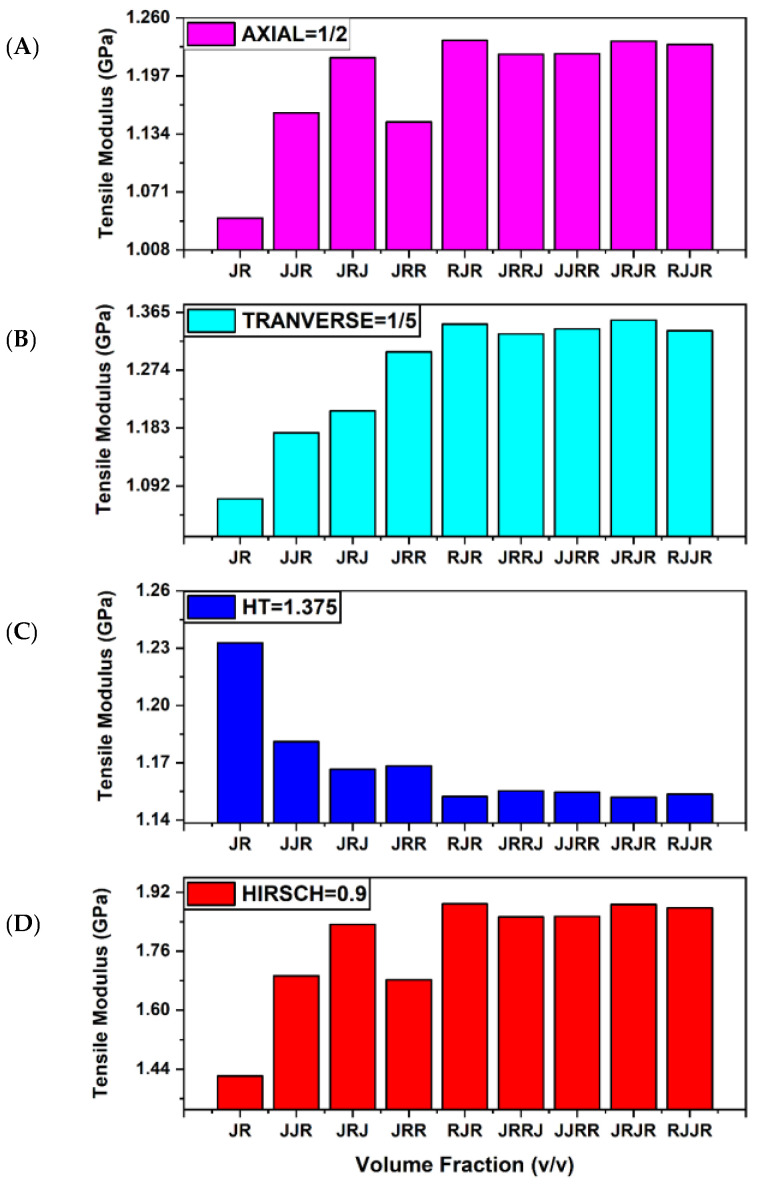
Modulus of composite hybrid: (**A**) ROM = 1/2 (**B**) IROM = 1/5, (**C**) HT = 1.375, (**D**) HI model = 0.9.

A parameter change is required to match the mathematical models with the current test results. All modifications are limited to the ROM, IROM, Halpin–Tsai, and Hirsch models. Tensile modulus predictions were made by first completing all known parameters and then entering all parameters into the model to match the data. The details of the parameter change are summarised in Table 8. Moreover, Figure 3 compares the experimental result and the value from a modified mathematical model.

To conform to the experimental results, the current micromechanical model must be modified. The 0.5 stress parameter refers to the ROM’s existing mathematical model. According to Osoka et al. (2018), the stress parameter of 0.5 is best suited for fibre reinforcement because it reinforces the stress parameter in two directions at right angles [27].

The IROM model employs a stress parameter of 0.2. According to Osoka et al. [27], the stress parameter of 0.2 is best suited for random and uniform fibre distributions in 3D space, which stresses in all directions. Although the stress parameter of 0.2 matches the experimental value, it does not accurately reflect the composite sample’s fibre arrangement and stress acting. The fibre arrangement is bidirectional, while the stress is only actuated vertically.

In the literature, the researcher employs various values for empiric value. Giner, Franco, and Vercher (2014) proposed an empirical value of 1.5 for the Halpin–Tsai model in their work [28]. A practical value of 1.5 is best suited for the random distribution of fibre with a volume fraction ranging from 22% to 55%, which is more realistic than the theoretical square array distribution. The 1.375 empiric value of the Halpin–Tsai model is best suited to the experimental value in the current work.

Because of the low contact, the low value of the stress parameter, as Sreenivasan et al. (2011) demonstrated, indicates low stress distinguishing between the fibre and the polymer content [29]. Regardless of the stress parameter, 0.01 and 0.9 do not match the current composite laminated result for the Hirsch model in the current study. According to Athijayamani et al. (2009), the stress parameter of 0.92 best represents the stress transfer between the randomly oriented fibre and the polymer matrix [30]. As a result, a hybrid woven composite does not adequately represent the Hirsch model. Note that the value obtained from the micromechanical model is not close to the experimental model, as shown in Table 9.

The woven hybrid composite is not well represented by the micromechanical models of ROM, IROM, HT, and HI.

## 4. Conclusions

This study aims to use a micromechanical model to forecast the behaviour of hybrid woven fabrics. The article discusses developing a micromechanical model for single woven and hybrid woven jute–ramie reinforced unsaturated polyester composites with varying layering sizes and stacking sequences compared to experimental data. According to the findings, HI’s single jute composite and micromechanics model are nearly identical to the measured value at volume fractions less than 15%. When the jute ramie volume fraction is greater than 20%, the experimental value follows the exact trend of HT = 1. The composite composed entirely of ramie fibres behaved differently than the composite composed entirely of jute fibres. For volume fractions ranging from 5% to 20%, the experiment value for a single ramie fibre is nearly identical to HT = 2. The data from the micromechanical model of the hybrid composite do not follow the experimental value trend. This is similar to the hybrid modified micromechanical model.

## Figures and Tables

**Table 1:** Design of hybrid jute–ramie reinforced unsaturated polyester composite.

		Jute	Ramie
Fabric thickness (mm)		1.23–1.59	1.14–1.82
Warp density (ends/2 cm)	df_1_	10	10
Weft density (ends/2 cm)	df_2_	10	10
Yarn spacing-Warp (mm)	P_1_	2	2
Yarn spacing-Weft (mm)	P_2_	2	2
Warp Yarn density (tex)	*N*_1_	326.2	314.9
Weft Yarn density (tex)	*N*_2_	316.3	442.4
Areal density (gm^−2^)	F_w_	324.35	385.45
Warp cover factor	C_1_	0.67	0.65
Weft cover factor	C_2_	0.66	0.77
Total cover factor	K	0.89	0.92
Fabric porosity	ε	0.18	0.22

**Table 2:** Design of hybrid jute–ramie reinforced unsaturated polyester composite.

No	Type of Composites	Jute wt. (g)	Ramie wt. (g)	Total wt. of Fibre (g)	Resin wt. (g)	Fibre wt. Fraction (w/w)	Fibre wt. Fraction (v/v)	Matrix wt. Fraction (w/w)
1	UPE	0.00	0.00	0.00	200.00	0.00	0.0	100.00
2	J	19.46	0.00	19.46	200.00	8.87	7.31	91.13
3	JJ	37.53	0.00	37.53	200.00	15.80	13.21	84.20
4	JJJ	64.98	0.00	64.98	200.00	24.52	20.85	75.48
5	JJJJ	82.12	0.00	82.12	200.00	29.10	24.97	70.89
6	R	0.00	21.54	21.54	200.00	9.72	7.93	90.28
7	RR	0.00	41.04	41.04	200.00	17.02	14.1	82.97
8	RRR	0.00	63.36	63.36	200.00	24.06	20.22	75.94
9	RRRR	0.00	84.39	84.39	200.00	29.67	25.24	70.33
10	JR	22.05	17.1	39.15	200.00	16.37	13.41	83.63
11	JJR	35.73	22.14	57.87	200.00	22.44	18.53	77.56
12	JRJ	45.18	21.69	66.87	200.00	25.06	20.79	74.94
13	RJR	23.22	44.46	67.68	200.00	25.28	20.89	74.72
14	JRRJ	38.33	43.65	81.98	200.00	29.07	24.09	70.93
15	JJRR	36.34	42.34	78.68	200.00	28.23	23.38	71.77
16	JRJR	35.79	43.71	79.5	200.00	28.44	23.55	71.56
17	RJJR	37.61	44.87	82.48	200.00	29.20	24.19	70.80

UPE: unsaturated polyester resin; J: jute, R: ramie.

**Table 3:** Tensile modulus for single jute fibre and ramie fibre.

	Tensile Modulus (GPa)								
Specimen	UPE	J	JJ	JJJ	JJJJ	R	RR	RRR	RRRR
1st	0.79	1.09	1.07	1.14	1.17	1.08	0.95	1.28	1.41
2nd	0.75	0.92	1.10	1.13	1.01	0.93	1.01	1.25	1.28
3rd	0.76	0.95	1.11	1.10	1.28	0.86	1.1	1.36	1.15
4th	0.78	1.12	1.02	1.07	1.37	1.06	1.14	1.24	1.43
5th	0.74	1.03	1.01	1.08	1.39	0.85	1.13	1.36	1.40
Average	0.77	1.02	1.06	1.11	1.24	0.96	1.08	1.30	1.33
Error	0.02	0.09	0.05	0.03	0.16	0.11	0.08	0.06	0.12

**Table 4:** Tensile modulus for hybrid jute–ramie composite.

	Tensile Modulus (GPa)								
Specimen	JR	JJR	JRJ	JRR	RJR	JRRJ	JJRR	JRJR	RJJR
1st	0.95	1.16	1.25	1.17	1.17	1.36	1.46	1.47	1.26
2nd	1.03	1.18	1.15	0.98	1.20	1.06	1.43	1.32	1.24
3rd	1.06	1.14	1.26	1.25	1.20	1.38	1.28	1.14	1.28
4th	1.11	1.19	1.22	1.10	1.18	1.09	1.29	1.45	1.30
5th	1.17	1.17	1.06	0.98	1.16	1.40	1.46	1.38	1.36
Average	1.06	1.17	1.19	1.10	1.18	1.26	1.38	1.35	1.29
Error	0.08	0.06	0.08	0.12	0.02	0.17	0.09	0.13	0.05

**Table 5:** Analysis of variance (ANOVA).

Source		Degree of Freedom	Adjusted Sum of Square	Adjusted Mean Square	F-Value	*p*-Value
Layering size	1st	4	0.37373	0.093433	27.90	0.00000284
Error	2nd	13	0.04353	0.003349		
Total	3rd	17	0.41727			

**Table 6:** Correlation between micromechanical data with experimental data for single jute composite.

Volume Content	ROM Axial	ROM Transverse	H-T EMP = 1	H-T EMP = 2	HI
0	0.00	0.00	0.00	0.00	0.00
7.31	0.16	−0.19	−0.13	−0.09	−0.05
13.21	0.44	−0.17	−0.06	0.01	0.07
20.85	0.77	−0.14	0.05	0.16	0.22
24.97	0.77	−0.19	0.02	0.15	0.19

**Table 7:** Correlation between micromechanical data with experimental data for single ramie composite.

Volume Content	ROM Axial	ROM Transverse	H-T EMP = 1	H-T EMP = 2	HI
0	0.00	0.00	0.00	0.00	0.00
7.93	0.11	−0.13	−0.08	−0.02	−0.04
14.1	0.19	−0.18	−0.09	0.02	−0.03
20.22	0.17	−0.27	−0.15	−0.02	−0.09
25.24	0.28	−0.24	−0.09	0.07	−0.03

**Table 8:** Stress parameter for modified micromechanical model.

	ROM	IROM	H-T	HI
	Stress parameter, *κ*	Stress parameter, *κ*	Stress parameter, *ξ*	Stress parameter, *κ*
Parameter value	1/2	1/5	1.25–1.5	0.9

**Table 9:** Correlation between modified micromechanical data with experimental data for hybrid composite.

	ROM = 1/2	IROM = 1/5	HT = 1.375	HI = 0.9
JR	−0.68	−0.65	−1.13	−0.38
JJR	−0.44	−0.38	−1.17	−0.44
JRJ	−0.32	−0.24	−1.18	−0.45
JRR	−0.44	−0.47	−1.18	−0.45
RJR	−0.26	−0.24	−1.19	−0.47
JRRJ	−0.30	−0.28	−1.19	−0.46

## Data Availability

Data are contained within the article.
